# Effect of poplar ecological retreat project on soil bacterial community structure in Dongting Lake wetland

**DOI:** 10.3389/fmicb.2022.1026872

**Published:** 2022-10-17

**Authors:** Haipeng Wu, Sha Xiao, Juan Dai, Ying Xiong, Jiao Cao, Xinyu Qu, Guiqiao Wang, Ruiqing Yang

**Affiliations:** ^1^School of Hydraulic and Environmental Engineering, Changsha University of Science and Technology, Changsha, China; ^2^Key Laboratory of Dongting Lake Aquatic Eco-Environmental Control and Restoration of Hunan Province, Changsha, China; ^3^Changjiang River Scientific Research Institute, Wuhan, China

**Keywords:** poplar ecological retreat, soil properties, bacterial community structure, Dongting Lake, ecological restoration

## Abstract

As an important environmental protection measure, the Poplar Ecological Retreat (PER) project aims to restore the ecology of the Dongting Lake (DL, China’s second largest freshwater lake) wetland. And its ecological impact is yet to be revealed. This study selected soil bacterial community structure (BCS) as an indicator of ecological restoration to explore the ecological impact of PER project on DL wetland. Soil samples were collected from reed area (RA, where poplar had never been planted, as the end point of ecological restoration for comparison in this study), poplar planting area (PA), poplar retreat for 1-year area (PR1A) and poplar retreat for 2 years area (PR2A), then their soil properties and BCS were measured. The results showed that the PER project caused significant changes in soil properties, such as the soil organic matter (SOM) and moisture, and an increase in the diversity and richness index of soil BCS. The Shannon-wiener index of RA, PA, PR1A and PR2A were 3.3, 2.63, 2.75 and 2.87, respectively. The number of operational taxonomic units (OTUs) changed similarly to the Shannon-wiener index. The Pearson correlation analysis and redundancy analysis (RDA) showed that the poplar retreat time, SOM and moisture content were the main factors leading to the increase of BCS diversity. All of these indicated that after the implementation of the PER project, the ecology of the lake area showed a trend of gradual recovery.

## Introduction

Monoculture of large-scale fast-growing tree plantations is considered to be one of the threats to biodiversity (Kindlmann et al., 2017; Egoh et al., 2020). Generally, they are superior to native species in competition for water, light and nutrients (Li et al., 2014b; Toledo et al., 2020), which may lead to transformation or even disappearance of natural ecosystems (Gardner et al., 2015; Sica et al., 2016). However，the use of fast-growing plantations is still widespread around the world to develop economies, meet landscape and other needs (Fashing et al., 2012). These plantations not only alter the structure, composition, and function of the natural ecosystem (Giejsztowt et al., 2019; Wang et al., 2020a), and would also affect the physicochemical and biological properties of the soil in various ways, such as altering the understory plant diversity (Li et al., 2014b), root and litter composition (Xu et al., 2014), depleting soil nutrients and water (Ferre et al., 2014), and intercepting and filtering surface runoff (Crampe et al., 2021). With the increasing attention on environmental issues in the country, it is of great significance to make further study on the impact of plantation forests on the ecosystem.

Dongting Lake (DL) includes three important international wetlands and four nature reserves (Ren et al., 2020). It provides water and soil conservation, carbon sequestration and other services (Sun et al., 2017; Deng et al., 2018), and plays an important role in sustaining regional ecological balance, regulating water resources of the Yangtze River system, and protecting biodiversity and rare species resources (Wang et al., 2020b; Hu et al., 2021). In the past century, however, the anthropogenic impacts and inconsequent land use have resulted in high level of ecological degradation in DL wetland (Sun et al., 2018; Wang et al., 2021). Poplar *(Populus deltoides)* as a common tree species for human afforestation, is a *Populus genus* of *Salicaceae* with moist-loving. It has a fast growth rate, strong adaptability and considerable economic benefits. In the 1970s, poplar trees began to be planted in Dongting Lake. With the rapid growth of planting area, poplar has become one of the important tree species for the construction of short-period industrial raw material forest in DL wetland (Li et al., 2014b). However, the single planting of large-scale fast-growing poplar plantation has led to various environmental problems, such as serious damage to the native vegetation of wetlands, decline of groundwater level, sharp changes in landscape pattern etc. (Li et al., 2014a; Sun et al., 2017; Lu et al., 2020). Coupled with the planting method of digging trenches and raising ridges, the service function of the wetland ecosystem was further destroyed (Du et al., 2011; Qu et al., 2022). These problems have caused the government to pay attention to the ecological restoration of the DL wetland (Jing et al., 2016). In order to alleviate a number of environmental problems caused by large-scale poplar planting, the “Poplar Ecological Retreat” (PER) project was launched in 2017 (Deng et al., 2022). About 6.5 million poplar trees had been retreated by the end of 2018, and all poplars in the core area of DL Nature Reserve had been removed (Bai et al., 2018; Hu et al., 2020). As a response to the governance, the wetland coverage and landscape pattern has transformed; and the regional ecological characteristics and functions, such as biodiversity, climate regulation, have also changed (Gong et al., 2019).

Several studies have been conducted on the PER project in the DL area. For example, Hu et al. (2020) founded that PER project improved the wetland water yield by changing landscape pattern; Qu et al. (2022) reported that habitat suitability of *Anatidae* and *Ardeidae* migratory birds could be significantly improved by PER project; The implementation of the PER project, the density of soil seed banks and the number of species increased significantly, and above-ground plant diversity gradually recovered (Deng et al., 2022). The PER project has considerably optimized the landscape pattern of DL wetland and promoted the improvement of the ecological environment in this area (Lu et al., 2020). Overall, these studies contributed to a comprehensive understanding of the ecological restoration of Dongting Lake wetland after the implementation of the PER project. Nevertheless, we know little about the PER project on soil biological characteristics, particularly the influence of the bacterial community structure (BCS).

Microorganisms have a great significance in soil structure formation, decomposition of SOM and minerals (Harris, 2009), maintaining system material transformation and energy flow (Hu et al., 2013), nitrogen fixation (Hartman et al., 2008), regulating growth of plants (Poret-Peterson et al., 2007), soil-borne diseases control (Wu et al., 2015) and other aspects. As the most active part of soil, microorganisms are extremely sensitive to human activities and external environmental changes compared with other large organisms, so as an evaluation index of soil quality has been widely recognized (Hargreaves et al., 2003; Card and Quideau, 2010; Wright and Wimberly, 2013). These means that the PER project will inevitably induce changes in soil microorganisms.

In this study, the soil BCS was applied as an indicator to investigate the ecological responses of the DL wetland to different time series of the implementation of the PER project. The purposes of this study were: (1) to analyze the response of the BCS and soil properties to different time series of the implementation of PER project in DL wetland; (2) to increase our knowledge of the relationship among human activities, soil properties and BCS in DL wetland; and (3) to provide scientific statistics and theoretical support for ecological restoration and environmental research in DL wetland.

## Study area and methods

### Study sites

**Figure 1:**
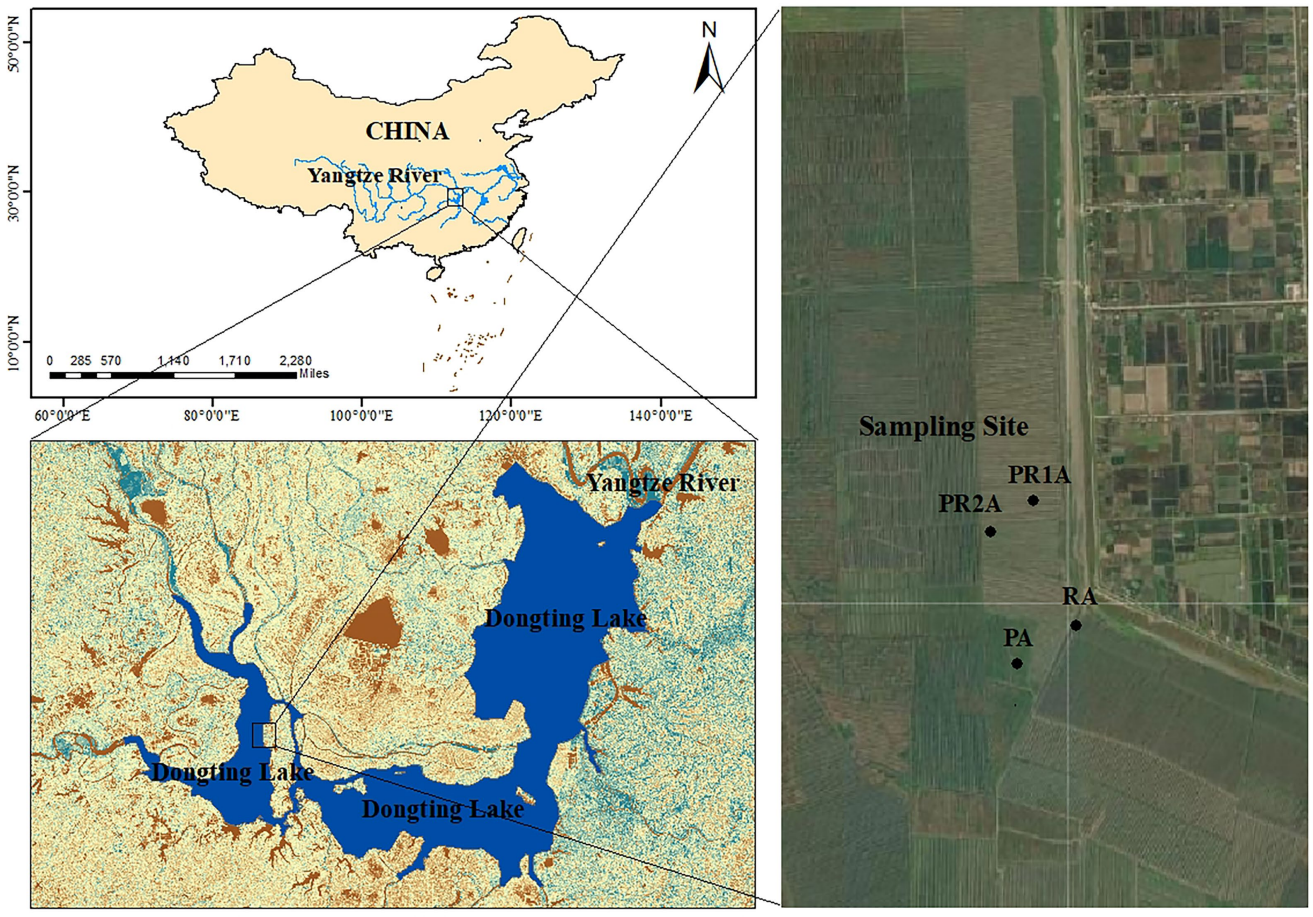
Locations of Dongting Lake and sample points of the reed area (RA), poplar planting area (PA), poplar retreat for 1-year area (PR1A) and poplar retreat for 2 years area (PR2A).

DL wetland (27°39′-29°51’N, 111°19′-113°34′E), located on the southern bank of Jingjiang River in the middle reaches of the Yangtze River (Dai et al., 2016). It receives water from the Yangtze River and its four tributaries (Xiangjiang, Zijiang, Yuanjiang and Lishui), then flows into the Yangtze River (Du et al., 2011). Its natural lake area is 2,625 km^2^, and the main Lake District is connected by floodways and numerous river networks (Ding and Li, 2011). This region has abundant rainfall and heat. The area of the lake and water level will change with the monsoon-driven precipitation, presented as the rainy season (May–October) with high area and water level and dry season (November – following April) with low area and water level (Wu et al., 2017). High water levels in the rainy season may cause poplars to be partially submerged, making it difficult to implement the PER project, so that forestry workers need to cut down poplar trees and transport them out during the dry season. Our sampling time was also scheduled for the dry season. In addition, the ravines originally dug need to be leveled for subsequent ecological restoration. Through the interpretation and comparison of remote sensing satellite images, the selected research area is located in the west DL. The specific location confirmed by field investigation is illustrated in Figure 1.

### Samples collection and soil properties determination

Soil samples at depths of 5 to 15 cm (Inacio et al., 2019) from 4 areas of DL wetland were collected in December 2020 and March 2021. Three parallel soil samples from each area were collected as the visible plant root matter and impurities were removed, and placed in plastic bags. Utilize a vehicle refrigerator to transport the samples to the laboratory. Samples for determination of soil properties were stored in a laboratory-specific refrigerator at 4°C, whereas samples for high-throughput sequencing were stored in an ultra-low temperature refrigerator at −50°C.

Soil organic matter (SOM) was measured by the combustion loss method, which means burning at 550°C for 4 h (Ghabbour et al., 2014). The quality method was used to determine the soil moisture, that is, the mass loss during overnight stoving at 105°C (Wu et al., 2015). Soil pH value was measured by portable pH meter (The ratio of dry soil to water is 1:2.5) (Damianova et al., 2016). The ammonium nitrogen was examined by indiophenol blue spectrophotometry (extracted with potassium chloride solution). The nitrate nitrogen was measured by phenol disulfonic acid spectrophotometry (Yan and Fu, 2021).

### High-throughput sequencing

Soil samples for molecular genome analysis were collected in March 2021. Extract DNA from 0.2 g of soil from three parallel samples in each region using the MagaBio Soil Genomic DNA Purification Kit. After that, Thermo NanoDrop One was used to detect the purity and concentration of the DNA, without dilution. The variable V4 region of the 16S rDNA coding genes was amplified by the bacterial universal primers GC 515F/806R. The PCR mixture containing 50 ng of template DNA, 2 × Premix Taq PCR MasterMix (TaKaRa Biotechnology Co., Dalian, China) 25 μl, 1 μl (10 μM) of each primer (-F and -R), then Nuclease-free water was injected to make the final volume 50 μl. The PCR amplification process was executed as follows: 30 cycles of denaturation were carried out after the initial denaturation (94°C, 5 min), including denaturation (94°C, 30 s), annealing (52°C, 30 s), extension (72°C, 30 s), and single elongation (72°C, 10 min), ended in a temperature of 4°C finally. Agarose gel (1%) electrophoresis was applied to detect the concentration and fragment length of PCR products, and the samples with the main band length between the normal range (290–310 bp) can be used for further experiments. After the PCR product concentration was compared by the GeneTools Analysis Software (SynGene, V.03.05.0,) we computed the volume needed for each sample in accordance with the equal quality principle, then mixed the PCR product. The PCR mixed products were recycled by Gel Extraction Kit (Omega, USA), and the target DNA fragments were eluted by the TE buffer solution. Follow the standard process to build the library, then the amplified sublibrary was sequenced by using Illumina Nova 6,000 platform of Magigene Biotechnology Co., Ltd. (Guangzhou, China).

### Bioinformatic analyses

Fastp (V.0.14.1) was used to execute window clipping of raw reads data on both ends (Kong, 2011), and effective clean tags were obtained. In accordance with the primer information of the head and tail of the sequence, the primer was deleted by cutadapt software, and the quality-controlled paired-end clean reads was gained. For dual-end sequencing data, usearch-Fastq_mergepairs (V.10, the preset minimum length of overlap is 16 bp, and the splicing sequence of the overlap area allows the maximum error to be 5 bp) was used to filter disconformed tags, then obtain the original stitching sequences (Raw Tags) on the basis of the overlap relationship among PE reads. Use the UCHIME algorithm in QIIME (V.2020.11.0) pipeline to remove chimeric sequences (Leray and Knowlton, 2016). Bacterial sequences were categorized into operational taxonomic units (OTUs) at a threshold value of 97% comparability by utilizing UPARSE (Edgar, 2013). Usearch-sintax (V.10.0.240) was used to compare the representative sequences of each OTU with UNITE (Abarenkov et al., 2010) and RDP (Cole et al., 2009) databases to obtain species annotation information (the default confidence threshold was set at 0.8), so as to understand the species origin of all sequences.

### Data processing and analysis

The alpha diversity of bacterial community was measured by Chao1 index and Shannon-wiener diversity index. After testing, all the data conform to the normal distribution, and the variance of each sample population is equal, so the mean values of physicochemical parameters of reed area (RA), poplar planting area (PA), poplar retreat for 1-year area (PR1A) and poplar retreat for 2 years area (PR2A) were compared with one-way analysis of variance (ANOVA), while the post-hoc Tukey’s test was applied to evaluate significant differences in the data (*p* < 0.05). Using Pearson correlation analysis to examine the single factor correlation between the parameters. The pluralistic relationships among the soil properties, the poplar retreat time and the BCS were confirmed by Canoco (V.5.0, Centre for Biometry, Wageningen, the Netherlands). Detrended correspondence analysis (DCA) showed that the length of the first sorting axis of DCA was 1.98, which indicated the linear response of species. Consequently, the default setting is used for redundancy analysis (RDA) to coordinate the soil characteristics and BCS. All analyses were performed by using SPSS (V.26) and R language.

## Results

### Responses of soil properties

Soil property parameters for each area are listed in Table 1. Several properties of soil samples from different regions have significant differences, especially SOM (*F* = 28.220, *p* < 0.001) and moisture (*F* = 20.861, *p* < 0.001). The SOM and moisture in PA were significantly lower than those in RA in December 2020 and March 2021. Compared with PA, the SOM and moisture in PR1A and PR2A increased, but still lower than that in RA. The pH, and the contents of nitrate nitrogen and ammonium nitrogen showed no significant differences among RA, PA, PR1A and PR2A.

**Table 1:** Values (mean ± SD) of soil properties of each site in December 2020 and March 2021.

Site.		Moisture	SOM	Ammonia nitrogen	Nitrate nitrogen	pH
RA	Dec20	45.20 ± 0.11ab	9.97 ± 0.30a	84.20 ± 0.53a	25.67 ± 0.38a	7.48 ± 0.09a
	Mar21	46.07 ± 2.27a	10.95 ± 0.62ab	85.12 ± 0.30a	26.87 ± 1.82a	7.51 ± 0.09a
PA	Dec20	33.73 ± 0.37 cd	4.96 ± 0.36d	83.24 ± 0.73a	24.27 ± 1.30a	7.47 ± 0.01a
	Mar21	35.98 ± 0.41 cd	5.90 ± 0.40d	85.27 ± 0.93a	30.30 ± 3.44a	7.52 ± 0.00a
PR1A	Dec20	36.53 ± 0.32 cd	6.26 ± 0.23 cd	86.15 ± 0.58a	23.47 ± 1.21a	7.51 ± 0.00a
	Mar21	38.43 ± 0.85c	7.95 ± 0.51bc	85.17 ± 0.84a	26.40 ± 0.70a	7.52 ± 0.01a
PR2A	Dec20	37.59 ± 0.32d	7.39 ± 0.31 cd	83.36 ± 0.32a	24.47 ± 1.23a	7.47 ± 0.01a
	Mar21	39.25 ± 0.36bc	8.44 ± 0.27c	85.24 ± 0.55a	26.67 ± 1.36a	7.49 ± 0.03a
*F*-value		20.861	28.220	2.361	1.588	1.885
Value of *p*		<0.001	<0.001	0.106	0.209	0.139

Different letters in same line indicate significantly difference (*p* < 0.05). SOM: soil organic matter.

Comparing the soil properties of each sample in December 2020 and March 2021, we found that there was no significant change in soil moisture, SOM, ammonium nitrogen, nitrate nitrogen and pH in RA and PA. The SOM and moisture increased in PR1A and PR2A from December 2020 to March 2021. The Pearson correlation coefficients among the factors considered listed in Table 2. The moisture of all soil samples was significantly positively correlated with SOM.

**Table 2:** Pearson’s correlation coefficients between the parameters of each site on March 2021.

	Moisture	SOM	pH	Ammonia nitrogen	Nitrate nitrogen	Number of OTUs	H
Moisture	1.000						
SOM	0.946^**^	1.000					
pH	−0.100^ns^	−0.097^ns^	1.000				
Ammonia nitrogen	0.295^ns^	0.236^ns^	0.371^ns^	1.000			
Nitrate nitrogen	−0.154^ns^	−0.220^ns^	0.045^ns^	−0.261^ns^	1.000		
Number of OTUs	0.866^*^	0.847^**^	−0.343^ns^	0.163^ns^	−0.114^ns^	1.000	
*H*	0.857^**^	0.864^**^	−0.179^ns^	0.288^ns^	−0.041^ns^	0.943^**^	1.000

H: Shannon-Wiener diversity index. Significance are indicated by ^***^p ≤ 0.001, ^**^p ≤ 0.01, ^*^p ≤ 0.05, while ns corresponds to no significant correlation (p > 0.05). SOM: soil organic matter.

### Response of bacterial community structure

After quality filtration, the 4 areas were sequenced to produce a total of 671,698 high-quality 16SrRNA gene sequences, then they were classified into 11,413 different OTUs. The shared bacterial OTUs among four areas was 3,072, which accounted for 39.8, 58.0, 49.6 and 47.3% of the total OTUs from RA, PA, PR1A and PR2A sites, respectively (Supplementary Figure S1). The top 15 most abundant taxa of the communities have no difference between regions at the phylum level (Supplementary Figure S2), while the *Proteobacteria*, *Actinobacteria*, *Bacteroides* and *Chloroflexi* were the dominant phyla. The relative abundance of *Bacteroidetes* in samples of PR2A (12.3%) was higher than PA (*p* < 0.001) and PR1A (*p* < 0.001). In the heat map, the top 30 most abundant taxa corresponding to 4 regions confirms the differences in bacterial community composition (Supplementary Figure S3).

**Figure 2:**
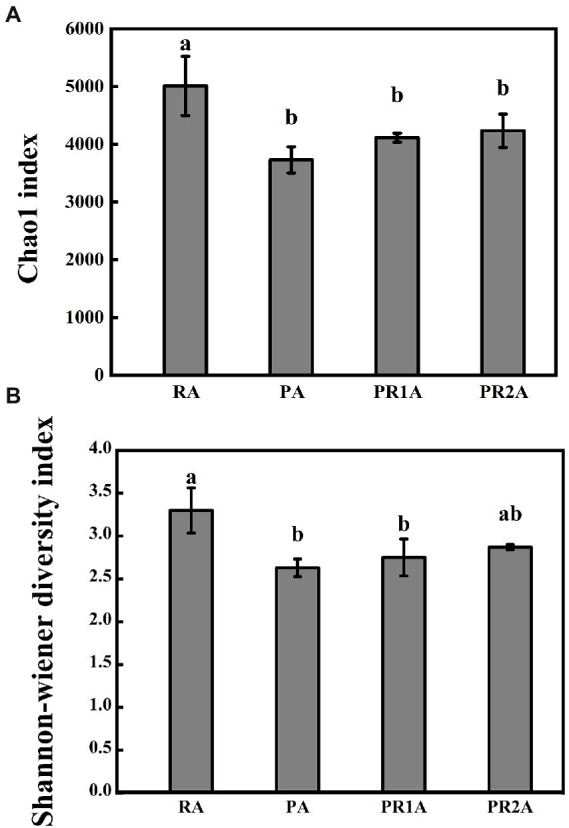
Chao1 index **(A)** and Shannon-Wiener diversity index **(B)** of the reed area (RA), poplar planting area (PA), poplar retreat for 1-year area (PR1A) and poplar retreat for 2 years area (PR2A) in March 2021. The vertical line above the histogram correspond to standard errors, while significant differences (*p* < 0.05) represented by different letters.

As shown in Figure 2, the chao1 index performed a significant difference (*F* = 8.655, *p* = 0.007), as the average value dropped from 5010.6 in RA to 3730.0 in PA, then rose to 4115.4 and 4156.5 in PR1A and PR2A. A significant difference (*F* = 7.947, *p* = 0.009) was also shown in Shannon-wiener index, as the average value decreased from 3.3 in RA to 2.63 in PA, then rose to 2.75 and 2.87 in PR1A and PR2A, respectively. The results of Chao1 and Shannon-Wiener index indicated that there were significant differences in BCS among soil samples from RA, PA, PR1A and PR2A. Overall, the BCS of RA performed more abundant and diverse than that of PA. 2 years after the implementation of the PER project, the BCS diversity has gradually increased, but it was still lower than that in RA.

**Figure 3:**
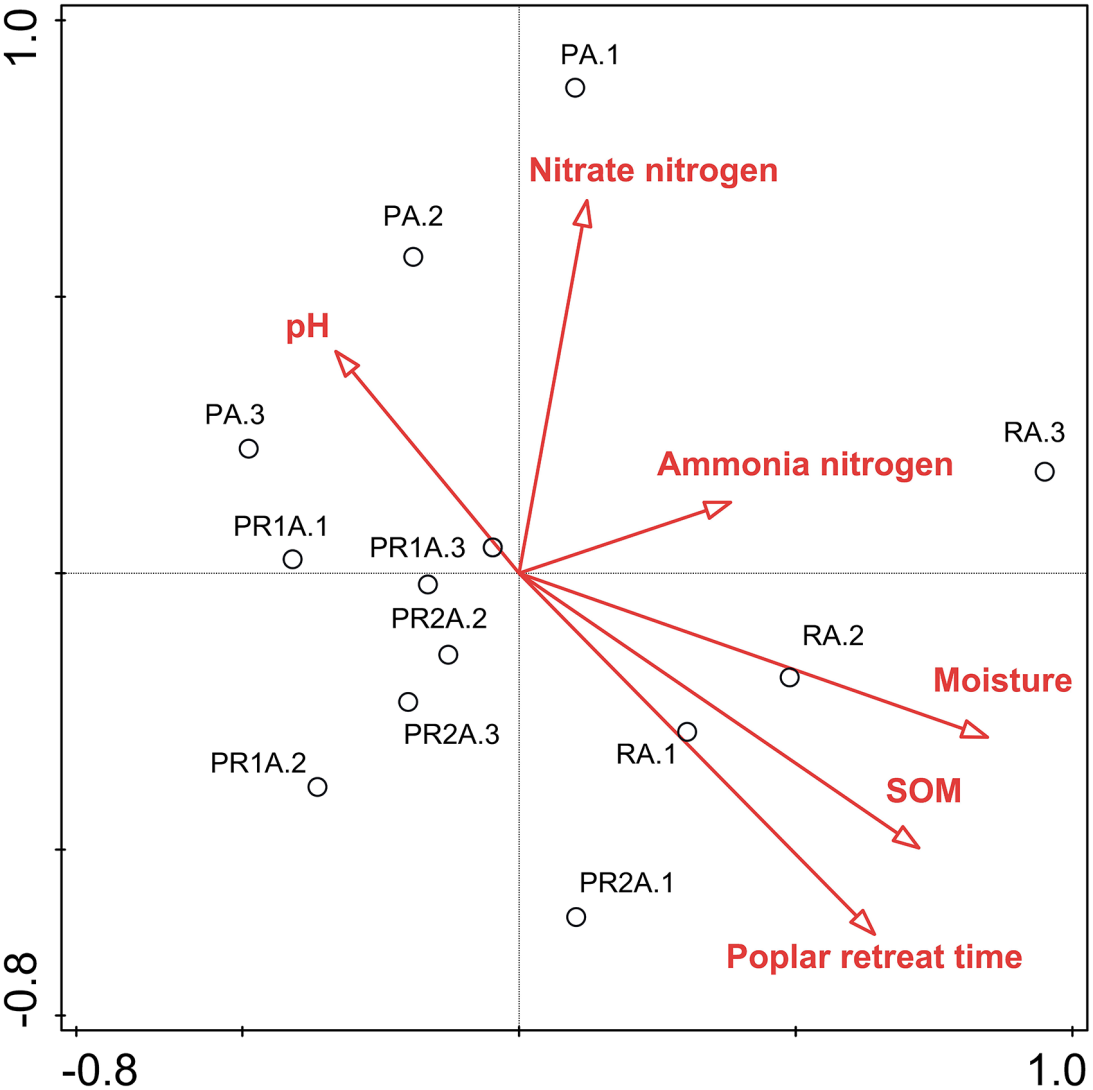
Two-dimensional ranking map of bacterial community structure and environmental factors based on Redundancy analysis. Samples are shown as circles; environmental factors are indicated by straight lines and hollow arrows. SOM: soil organic matter.

RDA biplots of the BCS and soil characteristics are illustrated in Figure 3. And Table 3 revealed that all soil properties parameters could explain 78.5% of the species variation. The poplar retreat time, the SOM and the moisture exerted extremely significant impacts on soil BCS. The order of the influence of these factors on BCS was: poplar retreat time > SOM > moisture > nitrate nitrogen > pH > ammonia nitrogen.

**Table 3:** Monte Carlo experiment to test the influence of poplar retreat time and soil properties on the results.

Parameters	% Variation explains	*p*-Value
Poplar retreat time	58.91	0.0158
SOM	54.76	0.0259
Moisture	49.91	0.0317
Nitrate nitrogen	21.42	0.2940
pH	14.27	0.4550
Ammonia nitrogen	9.40	0.5730
All above together	78.5	

SOM: soil organic matter.

## Discussion

### Response of soil properties

The results showed that, taking RA as control, the PER project had a significant influence on the soil properties. Reed has better carbon storage capacity than poplar, which may lead to lower SOM in PA than that in RA (Nocentini and Monti, 2017). Previous studies have shown that, compared with poplar, reed has a higher underground biomass development, more prosperous root system, and is more difficult to decompose than poplar litter (Monti and Zatta, 2009; Chimento and Amaducci, 2015), which makes more biological residues in the root system of reed and resulting in higher SOM. Moreover, understory plants were significantly correlated with the SOM (Abebe et al., 2020), the lack of understory plants would lead to low carbon storage in plantations (Lyu et al., 2021). The litter accumulated under the poplar plantation will cause direct physical obstacles to the germination of understory plants (Ellsworth et al., 2004; Yu et al., 2021a), and will also directly or indirectly affect the survival and renewal of understory plants by affecting the microenvironment such as light, temperature, and humidity, thereby reducing the SOM content (Graae and Heskjær, 1997; Esteso-Martínez and Gil-Pelegrín, 2004; Bo et al., 2018). Other studies have reported that poplar plantations could lower the water table and dry out the soil (Wilske et al., 2009; Boothroyd-Roberts et al., 2013), which is consistent with our research results. As shown in Table 1, the soil moisture in PA was significantly lower than that in RA. It can be speculated that the leaf area index of woody plants is usually higher than herbaceous plants, so that the stomatal conductance and transpiration rate of poplar are higher than reed (Wilske et al., 2009). While plant transpiration is considered to be the main mechanism of soil water efflux (Huang et al., 2011), which ultimately leads to the difference in soil moisture between PA and RA. Some studies have also shown that fast-growing plantations also have pumping effects in other ecosystems (Hernández-Santana et al., 2008; Tan et al., 2011). For example, Boothroyd-Roberts et al. (2013) found that due to transpiration, water content in plantation is lower than that in wasteland and secondary forest; Wilske et al. (2009) also reported that poplar dries the wetland soil by absorbing groundwater and transpiration. In this study, the SOM and moisture contents of samples in each region were in the order of PA < PR1A < PR2A < RA. This is because after the implementation of the PER project, the soil moisture by plantation transpiration loss no longer, and the growth of plants also have better environment conditions such as illumination, temperature, which cause the SOM and moisture to gradually increase in the process of natural restoration of wetlands (Figure 1).

Previous studies have reported that wildlife can also affect the properties of soil (Elhottová et al., 2012). Poplar blocked the landing of migratory birds, crowding out the habitat of wintering migratory birds (Qu et al., 2022). The poplar planting method of digging trenches and lifting ridges made the ground ravine crisscross, which leads to the destruction of fish spawning and migration channels (Li et al., 2020). As mentioned above, a lack of understory plants could also lead to a decrease in herbivores (Veblen et al., 2016). All of these destroyed the diversity of wildlife. After the implementation of the project, the living space of migratory birds, fish and wildlife was restored and the breeding conditions were better, resulting in an increase in their diversity and abundance, which would lead to changes in soil properties, such as SOM and moisture. This is because wildlife can import unstable carbon to the soil through the deposition of excrement, provoke new underground biomass and increase SOM (Bardgett et al., 1998; Olsen et al., 2011); they also change the rate of infiltration and drainage of soil water by compacting the soil and digging holes (Olsen et al., 2011). In a word, increased wildlife also improved SOM and water content in the soil.

Besides, Table 2 shows that there is a significant correlation between SOM and moisture, which consistent with the results obtained by Wu et al. (2015). This is because SOM improved the water holding capacity by ameliorating the colloid condition and improving the porosity of the soil (Douterelo et al., 2009). The higher the amount of SOM, the more stable and homogeneous the aggregate structure of the soil, and the more organic colloids are carried, thus adsorb and store more water (Dai et al., 2020). While soil moisture can affect plant uptake of mineral substance and alter the growth of plants by changing the concentration of soil mineral element ions, and ultimately the soil nutrient status (Kajiura et al., 2012); and can also affect SOM decomposition and carbon storage by altering soil redox conditions (Chen et al., 2018; Yu et al., 2021b). For example, Wu et al. (2013) and Qu et al. (2021) reported that SOM content would increase with soil moisture in a certain range; while Boothroyd-Roberts et al. (2013) and Lal (2020) founded that the recovery of SOM would also contribute to the increase of water content.

### Response of bacterial community structure

In this study, soil samples from areas that had never been planted with poplar (RA) showed higher BCS richness and diversity than the samples from areas that had been planted with poplar. After poplar retreat, the diversity of soil BCS increased gradually in the process of natural restoration of wetland. The PER project affects soil microbial community by changing soil properties. As mentioned above, PER projects affect soil properties by improving understory plant survival and regeneration conditions, reducing transpiration, restoring wildlife living spaces. Soil microorganism was extremely sensitive to the changes in soil properties, such as moisture and SOM. For example, water as a resource, solvent and transport medium can directly affect the structure of microbial communities (Schimel, 2018); and can also drive the growth and change of microbial communities by affecting the activity of various enzymes in the soil (Brockett et al., 2012; Zhang et al., 2021). In this study, the shortage of water caused by the planting of poplars resulted in a decrease in soil microbial abundance in soil samples of PA, which was attributed to limited dispersal of extracellular enzymes and nutrients in the soil due to water scarcity, thus inhibiting microbial growth (Wu et al., 2013; Yang et al., 2017). He et al. (2020) also reported that water deficiency would lead to a decrease in microbial abundance. After the implementation of the PER project, the restoration of wildlife populations and plant diversity injected more organic matter into the soil, resulting in an increase in the diversity of microbial community structures, as the SOM is an essential substrate for microbial survival (Marshman and Marshall, 1981; Yu et al., 2022). These soil characteristics can regulate nutrients and anaerobic or aerobic processes in the soil to affect microbial metabolism, and then change the community structure of microorganisms (Gutknecht et al., 2006; Card and Quideau, 2010). This study concluded that the SOM and the moisture imposed highly significant impacts on the soil BCS. Other studies also founded that the soil microbial diversity and structure could be affected by soil elements, such as SOM and moisture (Tang et al., 2011; Qu et al., 2021).

Noteworthy, taking the soil in the RA as the end point of the restoration for the control, the properties of the soil and the BCS diversity showed a gradual recovery trend after the implementation of the PER project. Though this change is not significant within 2 years, it can be speculated that with the extension of ecological restoration time, this change will further expand and reach a significant level.

After ecological restoration measures have achieved good results, how to further maintain the stability of the ecosystem is also an important topic. Material circulation is important for maintaining the stability of ecosystems. Studies have shown that the changes in soil BCS would directly affect the material circulation of the ecosystem (Jia et al., 2022; Zhu et al., 2022), so the changes of BCS caused by the PER project will also have an impact on the material circulation of DL wetland. Nevertheless, how the PER project will affect the material circulation of the DL ecosystem and what significance it has for maintaining the stability of the DL wetland ecosystem need to be further studied.

## Conclusion

The soil properties and BCS before and after the implementation of the PER project in DL wetlands was effectively estimated by means of high-throughput sequencing and methods of correlation and significance analysis. The study led to the following conclusions: the PER project has significantly changed the soil properties, especially the SOM and the moisture; there were also significant differences in BCS, the Chao1 index of PA, PR1A, PR2A and RA were 3730.0, 4115.4, 4156.5 and 5010.6, respectively. And the change trend of Shannon index is similar to Chao1 index, both of them increase steadily after the implementation of the project. The correlation analysis and redundancy analysis indicated that the difference in poplar retreat time, SOM and moisture content were the key influence of soil microbial community structure. The PER project would be the root cause for these changes.

The improvement of BCS diversity and richness indicates the effectiveness of PER project in ecological restoration of the DL wetland, suggesting that the PER project is a scientific demonstration for restoring the ecological environment of the DL wetland. In addition, these results also increase our knowledge of the relationship between human activities and environmental change in DL wetland, and provide scientific data and theoretical support for the ecological restoration of Dongting Lake wetland.

## Data availability statement

The datasets presented in this study can be found in online repositories. The names of the repository/repositories and accession number(s) can be found at: NCBI – PRJNA878642, SAMN30734537.

## Author contributions

HW: ideas, experimental resources, administrative support, funding support, writing review, academic supervision, and manuscript revision. SX: experimental design and implementation, material and equipment purchase, field research, data collection and analysis, and manuscript writing. JD and JC: concept, method, data auditing, and manuscript revision. YX: administrative support, funding support, and academic supervision. XQ and GW: software, field research, and manuscript revision. RY: field research, experiments, and manuscript revision. All authors contributed to the article and approved the submitted version.

## Funding

This research was financially supported by the National Natural Science Foundation of China (52270195, 51809011, and U21A2010), the Natural Science Foundation of Hunan Province (2021JJ40601), and the Scientific Research Project of the Education Department of Hunan province (20B005).

## Conflict of interest

The authors declare that the research was conducted in the absence of any commercial or financial relationships that could be construed as a potential conflict of interest.

## Publisher’s note

All claims expressed in this article are solely those of the authors and do not necessarily represent those of their affiliated organizations, or those of the publisher, the editors and the reviewers. Any product that may be evaluated in this article, or claim that may be made by its manufacturer, is not guaranteed or endorsed by the publisher.
