# Supplementary material for: Effect of poplar ecological retreat project on soil bacterial community structure in Dongting Lake wetland
Source: Front Microbiol. 2022 Oct 17;13:1026872. doi: 10.3389/fmicb.2022.1026872 (PMC9620962; doi:10.3389/fmicb.2022.1026872)
Supplement: Supplementary file 1 [file Data_Sheet_1.docx]

**The following are the supplementary figures related to this article:**


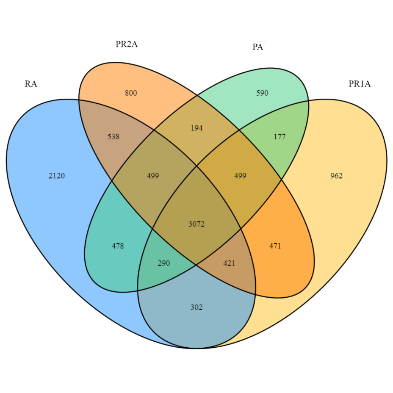


**Figure. S1.** Shared and unique OTUs from RA, PA, PR1A and PR2A.


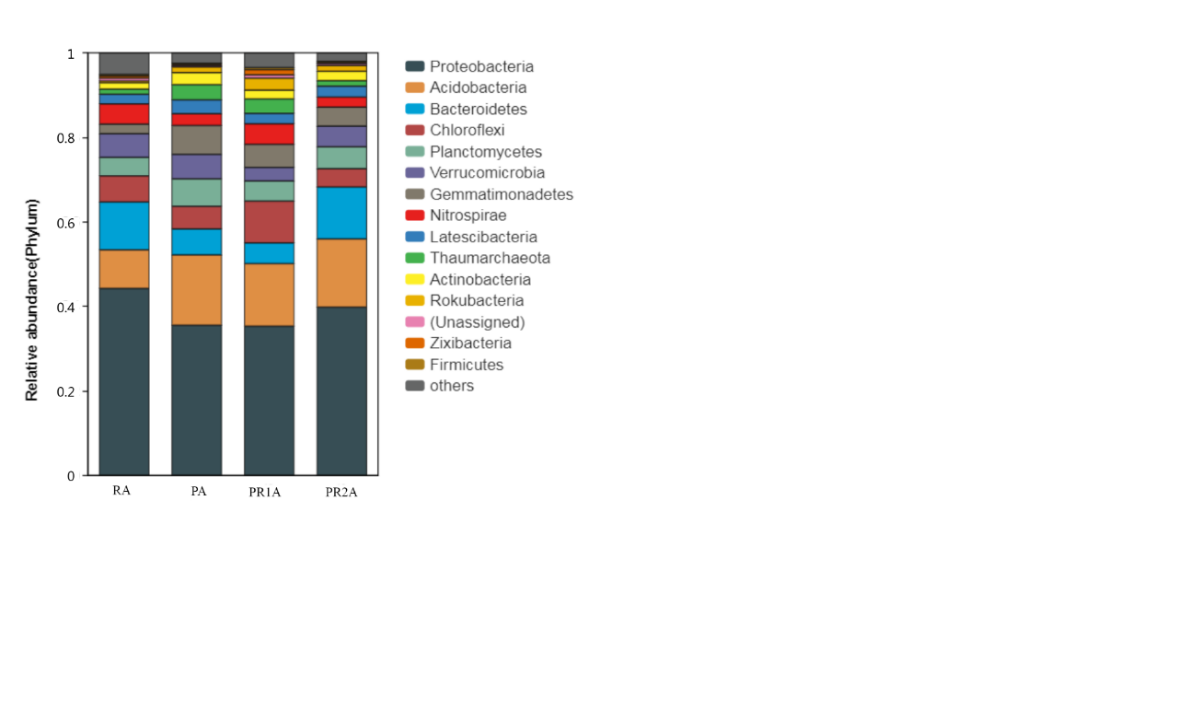


**Figure. S2.** Composition of bacteria in soil from RA, PA, PR1A and PR2A at the phylum level.


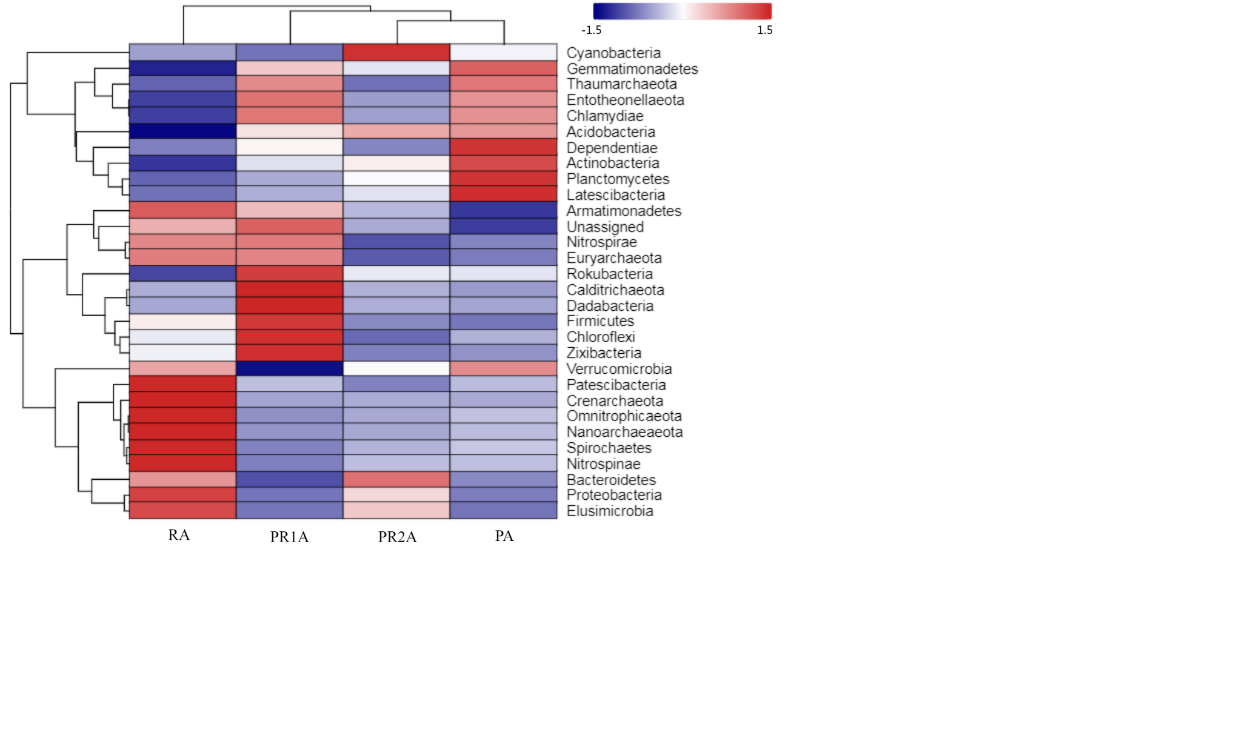


**Figure. S3.** Taxonomic composition of bacterial classes from RA, PA, PR1A and PR2A.
